# *N*-(1-Naphth­yl)benzene­sulfonamide

**DOI:** 10.1107/S1600536811039201

**Published:** 2011-10-05

**Authors:** Sifang Zhang, Yuewen Zhang, Chuntao Wang, Ruitao Zhu

**Affiliations:** a Department of Chemistry, Taiyuan Normal University, Taiyuan 030031, People’s Republic of China

## Abstract

In the title compound, C_16_H_13_NO_2_S, the C—SO_2_—NH—C torsion angle is −70.1 (2)°. The dihedral angle between the planes of the naphthyl ring system and the phenyl ring is 34.67 (4)°. In the crystal, mol­ecules are linked by inter­molecular N—H⋯O hydrogen bonds into chains along [100]. There are also π–π inter­actions between adjacent naphthyl groups [inter­planar spacing = 3.541 (3) Å] for mol­ecules stacked along [100].

## Related literature

For hydrogen-bonding modes of sulfonamides, see: Adsmond & Grant (2001 ▶). For related structures, see: Shakuntala *et al.* (2011 ▶). For standard bond-length data, see: Allen *et al.* (1987 ▶).
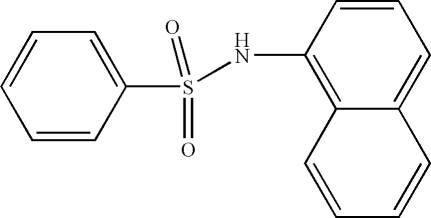


## Experimental

### 

#### Crystal data

C_16_H_13_NO_2_S
*M* *_r_* = 283.33
Orthorhombic, 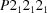

*a* = 4.9232 (5) Å
*b* = 15.4162 (15) Å
*c* = 18.2102 (17) Å
*V* = 1382.1 (2) Å^3^
*Z* = 4
Mo *K*α radiation
μ = 0.23 mm^−1^
*T* = 298 K
0.43 × 0.33 × 0.32 mm

#### Data collection

Bruker SMART CCD area-detector diffractometer
Absorption correction: multi-scan (*SADABS*; Bruker, 2007 ▶) *T*_min_ = 0.906, *T*_max_ = 0.929
6917 measured reflections
2438 independent reflections
2178 reflections with *I* > σ(*I*)
*R*_int_ = 0.032

#### Refinement

*R*[*F* ^2^ > 2σ(*F* ^2^)] = 0.035
*wR*(*F* ^2^) = 0.084
*S* = 1.09
2438 reflections
182 parameters
H-atom parameters constrained
Δρ_max_ = 0.18 e Å^−3^
Δρ_min_ = −0.20 e Å^−3^
Absolute structure: Flack (1983 ▶), 983 Friedel pairs
Flack parameter: 0.06 (9)

### 

Data collection: *SMART* (Bruker, 2007 ▶); cell refinement: *SAINT* (Bruker, 2007 ▶); data reduction: *SAINT*; program(s) used to solve structure: *SHELXS97* (Sheldrick, 2008 ▶); program(s) used to refine structure: *SHELXL97* (Sheldrick, 2008 ▶); molecular graphics: *SHELXTL* (Sheldrick, 2008 ▶); software used to prepare material for publication: *SHELXTL*.

## Supplementary Material

Crystal structure: contains datablock(s) I, global. DOI: 10.1107/S1600536811039201/pk2348sup1.cif

Structure factors: contains datablock(s) I. DOI: 10.1107/S1600536811039201/pk2348Isup2.hkl

Supplementary material file. DOI: 10.1107/S1600536811039201/pk2348Isup3.cml

Additional supplementary materials:  crystallographic information; 3D view; checkCIF report

## Figures and Tables

**Table 1:** Hydrogen-bond geometry (Å, °)

*D*—H⋯*A*	*D*—H	H⋯*A*	*D*⋯*A*	*D*—H⋯*A*
N1—H1⋯O1^i^	0.90	2.05	2.911 (3)	159

Symmetry code: (i) 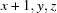.

**Fig. 1.**
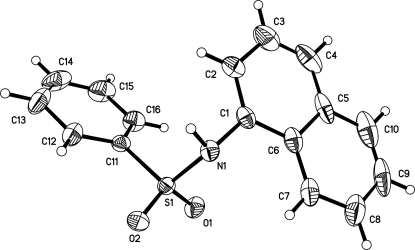
The molecular structure of the title compound with displacement ellipsoids are drawn at the 30% probability level.

**Fig. 2.**
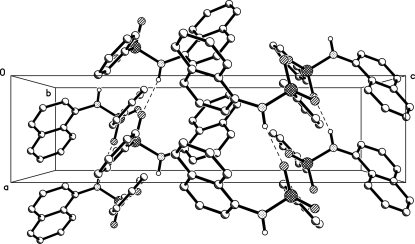
Part of the crystal structure with hydrogen bonds drawn as dashed lines. Only H atoms involved in hydrogen bonds are shown.

## supplementary crystallographic information

### Comment

Sulfonamide moieties are constituents of many biologically important compounds.  The hydrogen bonding preferences of sulfonamides has been investigated (Adsmond & Grant, 2001). In this paper, we present the crystal structure of the title compound.

The molecular structure of is shown in Fig. 1.  The bond lengths (Allen *et al.*, 1987) and angles are normal.  The molecule is twisted at the S atom with C—SO_2_—NH—C torsion angle of -70.14 (2) °.  The dihedral between the planes of the naphthyl and benzene groups is 34.67 (4) °.  In the crystal, molecules are linked by intermolecular N—H···O hydrogen bonds into chains along [100].  There are also π-π interactions between adjacent naphthyl groups (interplanar spacing 3.541 (3) Å) for molecules stacked along [100].

### Experimental

To a 100 ml round flask fitted with a condenser was added 1-naphthylamine (1.43 g, 10 mmol), dichloromethane (15 ml) and triethylamine(0.5 ml) with magnetic stirring.  Benzenesulfonyl chloride (1.76 g, 10 mmol) was added gradually.  The reaction mixture was stirred at room temperature for 1 h and then refluxed for 2 h.  The product precipitated as a white powder, which was washed three times with water and dichloromethane.  Recrystallization from ethyl alcohol produced the crystals of the title compound.

### Refinement

H atoms were placed in idealized positions and allowed to ride on their respective parent atoms, with C—H = 0.93 Å, N—H = 0.90Å and *U*_iso_(H)= 1.2*U*_eq_(C,*N*).

### Crystal data

**Table:**

C_16_H_13_NO_2_S	*F*(000) = 592
*M**_r_* = 283.33	*D*_x_ = 1.362 Mg m^−^^3^
Orthorhombic, *P*2_1_2_1_2_1_	Mo *K*α radiation, λ = 0.71073 Å
Hall symbol: P 2ac 2ab	Cell parameters from 3370 reflections
*a* = 4.9232 (5) Å	θ = 2.6–26.0°
*b* = 15.4162 (15) Å	µ = 0.23 mm^−^^1^
*c* = 18.2102 (17) Å	*T* = 298 K
*V* = 1382.1 (2) Å^3^	Block-like, colorless
*Z* = 4	0.43 × 0.33 × 0.32 mm

### Data collection

**Table:**

Bruker SMART CCD area-detector diffractometer	2438 independent reflections
Radiation source: fine-focus sealed tube	2178 reflections with *I* > σ(*I*)
graphite	*R*_int_ = 0.032
φ and ω scans	θ_max_ = 25.0°, θ_min_ = 2.6°
Absorption correction: multi-scan (*SADABS*; Bruker, 2007)	*h* = −5→5
*T*_min_ = 0.906, *T*_max_ = 0.929	*k* = −17→18
6917 measured reflections	*l* = −14→21

### Refinement

**Table:**

Refinement on *F*^2^	Hydrogen site location: inferred from neighbouring sites
Least-squares matrix: full	H-atom parameters constrained
*R*[*F*^2^ > 2σ(*F*^2^)] = 0.035	*w* = 1/[σ^2^(*F*_o_^2^) + (0.037*P*)^2^ + 0.2812*P*] where *P* = (*F*_o_^2^ + 2*F*_c_^2^)/3
*wR*(*F*^2^) = 0.084	(Δ/σ)_max_ < 0.001
*S* = 1.09	Δρ_max_ = 0.18 e Å^−^^3^
2438 reflections	Δρ_min_ = −0.20 e Å^−^^3^
182 parameters	Extinction correction: *SHELXL97* (Sheldrick, 2008), Fc^*^=kFc[1+0.001xFc^2^λ^3^/sin(2θ)]^-1/4^
0 restraints	Extinction coefficient: 0.038 (2)
Primary atom site location: structure-invariant direct methods	Absolute structure: Flack (1983), 983 Friedel pairs
Secondary atom site location: difference Fourier map	Flack parameter: 0.06 (9)

### Special details

**Table:**

Geometry. All e.s.d.'s (except the e.s.d. in the dihedral angle between two l.s. planes) are estimated using the full covariance matrix. The cell e.s.d.'s are taken into account individually in the estimation of e.s.d.'s in distances, angles and torsion angles; correlations between e.s.d.'s in cell parameters are only used when they are defined by crystal symmetry. An approximate (isotropic) treatment of cell e.s.d.'s is used for estimating e.s.d.'s involving l.s. planes.
Refinement. Refinement of *F*^2^ against ALL reflections. The weighted *R*-factor *wR* and goodness of fit *S* are based on *F*^2^, conventional *R*-factors *R* are based on *F*, with *F* set to zero for negative *F*^2^. The threshold expression of *F*^2^ > σ(*F*^2^) is used only for calculating *R*-factors(gt) *etc*. and is not relevant to the choice of reflections for refinement. *R*-factors based on *F*^2^ are statistically about twice as large as those based on *F*, and *R*- factors based on ALL data will be even larger.

### Fractional atomic coordinates and isotropic or equivalent isotropic displacement parameters (Å^2^)

**Table:**

	*x*	*y*	*z*	*U*_iso_*/*U*_eq_	
S1	0.63000 (11)	0.47539 (3)	0.28894 (3)	0.03525 (17)	
N1	0.7982 (4)	0.50023 (12)	0.36336 (10)	0.0388 (5)	
H1	0.9726	0.4850	0.3559	0.047*	
O1	0.3486 (3)	0.48108 (11)	0.30720 (9)	0.0496 (4)	
O2	0.7374 (3)	0.52862 (11)	0.23221 (9)	0.0486 (4)	
C1	0.7144 (5)	0.46260 (17)	0.43237 (13)	0.0430 (6)	
C2	0.8252 (6)	0.38695 (18)	0.45628 (15)	0.0574 (7)	
H2	0.9547	0.3589	0.4277	0.069*	
C3	0.7467 (9)	0.3508 (2)	0.52341 (18)	0.0800 (11)	
H3	0.8277	0.2999	0.5400	0.096*	
C4	0.5555 (9)	0.3894 (3)	0.56351 (18)	0.0824 (12)	
H4	0.4994	0.3634	0.6070	0.099*	
C5	0.4352 (6)	0.4686 (3)	0.54214 (14)	0.0673 (9)	
C6	0.5182 (5)	0.50785 (18)	0.47415 (13)	0.0503 (7)	
C7	0.4088 (6)	0.5887 (2)	0.45330 (16)	0.0617 (8)	
H7	0.4671	0.6155	0.4103	0.074*	
C8	0.2160 (7)	0.6281 (3)	0.4966 (2)	0.0894 (12)	
H8	0.1439	0.6815	0.4831	0.107*	
C9	0.1278 (8)	0.5871 (4)	0.5617 (2)	0.1007 (15)	
H9	−0.0060	0.6132	0.5902	0.121*	
C10	0.2347 (8)	0.5107 (4)	0.5831 (2)	0.0960 (14)	
H10	0.1733	0.4853	0.6264	0.115*	
C11	0.6966 (5)	0.36643 (14)	0.26609 (12)	0.0375 (6)	
C12	0.8977 (6)	0.34830 (16)	0.21627 (15)	0.0541 (6)	
H12	0.9960	0.3927	0.1942	0.065*	
C13	0.9509 (7)	0.2619 (2)	0.19960 (19)	0.0767 (10)	
H13	1.0869	0.2477	0.1663	0.092*	
C14	0.7995 (8)	0.1971 (2)	0.2331 (2)	0.0815 (12)	
H14	0.8348	0.1393	0.2220	0.098*	
C15	0.6022 (8)	0.21681 (18)	0.28156 (19)	0.0747 (9)	
H15	0.5027	0.1725	0.3034	0.090*	
C16	0.5467 (6)	0.30098 (16)	0.29891 (15)	0.0553 (7)	
H16	0.4100	0.3143	0.3323	0.066*	

### Atomic displacement parameters (Å^2^)

**Table:**

	*U*^11^	*U*^22^	*U*^33^	*U*^12^	*U*^13^	*U*^23^
S1	0.0321 (3)	0.0373 (3)	0.0364 (3)	0.0023 (3)	0.0012 (2)	0.0003 (3)
N1	0.0274 (10)	0.0482 (11)	0.0407 (10)	−0.0036 (7)	0.0033 (8)	−0.0040 (8)
O1	0.0304 (8)	0.0613 (10)	0.0570 (10)	0.0060 (9)	−0.0004 (8)	−0.0087 (9)
O2	0.0576 (10)	0.0435 (8)	0.0448 (9)	0.0013 (9)	0.0034 (8)	0.0097 (8)
C1	0.0377 (13)	0.0569 (15)	0.0344 (12)	−0.0124 (12)	−0.0021 (10)	−0.0027 (11)
C2	0.0598 (18)	0.0633 (17)	0.0491 (15)	−0.0032 (14)	−0.0107 (14)	0.0038 (13)
C3	0.105 (3)	0.079 (2)	0.055 (2)	−0.016 (2)	−0.009 (2)	0.0166 (17)
C4	0.101 (3)	0.104 (3)	0.0423 (18)	−0.044 (2)	−0.0106 (19)	0.0179 (18)
C5	0.0537 (18)	0.114 (3)	0.0340 (13)	−0.0348 (19)	0.0049 (13)	−0.0198 (17)
C6	0.0384 (13)	0.0705 (18)	0.0420 (13)	−0.0158 (13)	0.0000 (11)	−0.0140 (12)
C7	0.0512 (18)	0.083 (2)	0.0513 (16)	−0.0034 (15)	0.0029 (14)	−0.0252 (15)
C8	0.067 (2)	0.119 (3)	0.082 (2)	0.020 (2)	−0.0036 (19)	−0.046 (2)
C9	0.058 (2)	0.178 (4)	0.066 (2)	−0.009 (3)	0.020 (2)	−0.067 (3)
C10	0.070 (2)	0.161 (4)	0.057 (2)	−0.042 (3)	0.0155 (18)	−0.034 (3)
C11	0.0370 (13)	0.0366 (11)	0.0389 (12)	0.0012 (10)	−0.0069 (10)	−0.0002 (9)
C12	0.0507 (15)	0.0522 (13)	0.0594 (15)	0.0053 (13)	0.0022 (15)	−0.0121 (13)
C13	0.064 (2)	0.075 (2)	0.091 (2)	0.0250 (17)	−0.0058 (19)	−0.0371 (19)
C14	0.089 (3)	0.0419 (15)	0.113 (3)	0.0147 (17)	−0.046 (2)	−0.0218 (18)
C15	0.092 (2)	0.0398 (14)	0.092 (2)	−0.0081 (16)	−0.021 (2)	0.0030 (16)
C16	0.0582 (17)	0.0461 (14)	0.0615 (17)	−0.0063 (12)	−0.0032 (15)	0.0029 (13)

### Geometric parameters (Å, °)

**Table:**

S1—O2	1.4214 (16)	C7—H7	0.9300
S1—O1	1.4273 (17)	C8—C9	1.411 (6)
S1—N1	1.6337 (19)	C8—H8	0.9300
S1—C11	1.761 (2)	C9—C10	1.348 (6)
N1—C1	1.444 (3)	C9—H9	0.9300
N1—H1	0.9000	C10—H10	0.9300
C1—C2	1.359 (4)	C11—C12	1.372 (3)
C1—C6	1.414 (4)	C11—C16	1.386 (3)
C2—C3	1.398 (4)	C12—C13	1.391 (4)
C2—H2	0.9300	C12—H12	0.9300
C3—C4	1.332 (5)	C13—C14	1.388 (5)
C3—H3	0.9300	C13—H13	0.9300
C4—C5	1.411 (5)	C14—C15	1.347 (5)
C4—H4	0.9300	C14—H14	0.9300
C5—C10	1.397 (5)	C15—C16	1.363 (4)
C5—C6	1.437 (4)	C15—H15	0.9300
C6—C7	1.410 (4)	C16—H16	0.9300
C7—C8	1.376 (4)		
			
O2—S1—O1	119.67 (11)	C6—C7—H7	120.0
O2—S1—N1	106.20 (10)	C7—C8—C9	119.8 (4)
O1—S1—N1	106.52 (10)	C7—C8—H8	120.1
O2—S1—C11	108.04 (11)	C9—C8—H8	120.1
O1—S1—C11	107.11 (11)	C10—C9—C8	121.0 (3)
N1—S1—C11	108.98 (10)	C10—C9—H9	119.5
C1—N1—S1	118.87 (14)	C8—C9—H9	119.5
C1—N1—H1	107.4	C9—C10—C5	121.8 (4)
S1—N1—H1	107.3	C9—C10—H10	119.1
C2—C1—C6	121.7 (2)	C5—C10—H10	119.1
C2—C1—N1	120.6 (2)	C12—C11—C16	121.4 (2)
C6—C1—N1	117.7 (2)	C12—C11—S1	119.00 (18)
C1—C2—C3	120.8 (3)	C16—C11—S1	119.57 (19)
C1—C2—H2	119.6	C11—C12—C13	118.4 (3)
C3—C2—H2	119.6	C11—C12—H12	120.8
C4—C3—C2	119.7 (3)	C13—C12—H12	120.8
C4—C3—H3	120.1	C14—C13—C12	119.5 (3)
C2—C3—H3	120.1	C14—C13—H13	120.3
C3—C4—C5	122.2 (3)	C12—C13—H13	120.3
C3—C4—H4	118.9	C15—C14—C13	120.9 (3)
C5—C4—H4	118.9	C15—C14—H14	119.6
C10—C5—C4	123.4 (4)	C13—C14—H14	119.6
C10—C5—C6	117.7 (4)	C14—C15—C16	120.7 (3)
C4—C5—C6	118.8 (3)	C14—C15—H15	119.6
C7—C6—C1	123.5 (2)	C16—C15—H15	119.6
C7—C6—C5	119.7 (3)	C15—C16—C11	119.1 (3)
C1—C6—C5	116.8 (3)	C15—C16—H16	120.4
C8—C7—C6	119.9 (3)	C11—C16—H16	120.4
C8—C7—H7	120.0		
			
O2—S1—N1—C1	173.69 (18)	C5—C6—C7—C8	−2.5 (4)
O1—S1—N1—C1	45.1 (2)	C6—C7—C8—C9	−0.2 (5)
C11—S1—N1—C1	−70.1 (2)	C7—C8—C9—C10	1.6 (6)
S1—N1—C1—C2	91.7 (2)	C8—C9—C10—C5	−0.3 (6)
S1—N1—C1—C6	−89.3 (2)	C4—C5—C10—C9	178.8 (3)
C6—C1—C2—C3	0.4 (4)	C6—C5—C10—C9	−2.4 (5)
N1—C1—C2—C3	179.3 (3)	O2—S1—C11—C12	19.9 (2)
C1—C2—C3—C4	2.0 (5)	O1—S1—C11—C12	150.09 (19)
C2—C3—C4—C5	−2.8 (5)	N1—S1—C11—C12	−95.1 (2)
C3—C4—C5—C10	−179.9 (3)	O2—S1—C11—C16	−160.45 (19)
C3—C4—C5—C6	1.2 (4)	O1—S1—C11—C16	−30.3 (2)
C2—C1—C6—C7	176.5 (2)	N1—S1—C11—C16	84.6 (2)
N1—C1—C6—C7	−2.4 (3)	C16—C11—C12—C13	−0.6 (4)
C2—C1—C6—C5	−1.9 (3)	S1—C11—C12—C13	179.0 (2)
N1—C1—C6—C5	179.2 (2)	C11—C12—C13—C14	0.4 (4)
C10—C5—C6—C7	3.7 (4)	C12—C13—C14—C15	−0.1 (5)
C4—C5—C6—C7	−177.4 (3)	C13—C14—C15—C16	−0.1 (5)
C10—C5—C6—C1	−177.9 (2)	C14—C15—C16—C11	−0.1 (5)
C4—C5—C6—C1	1.1 (4)	C12—C11—C16—C15	0.5 (4)
C1—C6—C7—C8	179.2 (3)	S1—C11—C16—C15	−179.2 (2)

### Hydrogen-bond geometry (Å, °)

**Table:**

*D*—H···*A*	*D*—H	H···*A*	*D*···*A*	*D*—H···*A*
N1—H1···O1^i^	0.90	2.05	2.911 (3)	159.

Symmetry codes: (i) *x*+1, *y*, *z*.
